# Effect of Luting Materials on the Accuracy of Fit of Zirconia Copings: A Non-Destructive Digital Analysis Method

**DOI:** 10.3390/ma17092130

**Published:** 2024-05-01

**Authors:** Lara Berger, Ragai-Edward Matta, Christian Markus Weiß, Werner Adler, Manfred Wichmann, José Ignacio Zorzin

**Affiliations:** 1 Department of Prosthodontics, University Hospital Erlangen, Glückstrasse 11, 91054 Erlangen, Germany; lara.berger@uk-erlangen.de (L.B.); weiss_christian_markus@outlook.de (C.M.W.);; 2 Institute of Medical Informatics, Biometry and Epidemiology (IMBE) of the Friedrich-Alexander-University, Erlangen-Nuremberg, Waldstrasse 6, 91054 Erlangen, Germany; werner.adler@fau.de; 3 Dental Clinic 1—Department of Operative Dentistry and Periodontology, Erlangen University Hospital, Glueckstrasse 11, 91054 Erlangen, Germany; jose.zorzin@fau.de

**Keywords:** marginal fit, zirconia, self-adhesive resin cement, glass–ionomer cement, resin-modified glass–ionomer cement, zinc phosphate cement, CAD–CAM technology, ceramics, dental materials, prosthodontics

## Abstract

The marginal accuracy of fit between prosthetic restorations and abutment teeth represents an essential aspect with regard to long-term clinical success. Since the final gap is also influenced by the luting techniques and materials applied, this study analyzed the accuracy of the fit of single-tooth zirconia copings before and after cementation using different luting materials. Forty plaster dies with a corresponding zirconia coping were manufactured based on a single tooth chamfer preparation. The copings were luted on the plaster dies (*n* = 10 per luting material) with a zinc phosphate (A), glass–ionomer (B), self-adhesive resin (C), or resin-modified glass–ionomer cement (D). The accuracy of fit for each coping was assessed using a non-destructive digital method. Intragroup statistical analysis was conducted using Wilcoxon signed rank tests and intergroup analysis by Kruskal–Wallis and Mann–Whitney U tests (α = 0.05). Accuracy of fit was significantly different before/after cementation within A (0.033/0.110 µm) and B (0.035/0.118 µm; *p* = 0.002). A had a significantly increased marginal gap compared to C and D, and B compared to C and D (*p* ≤ 0.001). Significantly increased vertical discrepancies between A and B versus C and D (*p* < 0.001) were assessed. Of the materials under investigation, the zinc phosphate cement led to increased vertical marginal discrepancies, whereas the self-adhesive resin cement did not influence the restoration fit.

## 1. Introduction

Indirect all-ceramic restorations can realistically imitate natural human teeth, and therefore enjoy a very high popularity among dentists as they satisfy the increasing aesthetic demands of patients nowadays [1,2]. In this context, the spectrum of treatment methods and processing technologies must be continuously improved in order to optimally combine optimized functionality, biocompatibility, and the aesthetics of these ceramic materials [3,4].

There is a general digital transformation occurring within everyday dental practice, accompanied by an increasing interest in computer-assisted processes for the fabrication of dental prostheses in order to offer a standardized manufacturing chain with improved technical and biological properties of the component [5]. Considering this, the Computer-Aided Design (CAD)/Computer-Aided Manufacturing (CAM) fabrication of ceramic restorations is usually carried out by subtractive processes in which the workpiece is milled out of an industrially prefabricated blank [3]. The subtractive milling process is an advanced technique for the fabrication of ceramic restorations that has been proven over more than two decades [3,6,7]. CAD/CAM technology was pioneered and introduced to dentistry by François Duret in 1971 with his theoretical and experimental research on the computer-assisted manufacturing crowns. In 1980, Mörmann and Brandestini started with the development of a CAD/CAM system with an intraoral camera, a design computer and a milling unit using a ceramic block for manufacturing inlays at chair-side. Their research led in 1985 to the CEREC system (Dentsply Sirona, Bensheim, Germany). Based on these technologies, further chair- and lab-side dental CAD/CAM systems were developed [8]. The manufacturing of dental prostheses can also be performed using additive processes. In the field of dentistry, there are two main technologies that are widely used. One of them is stereolithography (SLA), which is typically utilized to create models, aligners, and provisional structures. The other is direct metal laser sintering (DMLS), which has the capability to produce metal dental crowns and appliance frames [9].

In contemporary dental practices, silicate and oxide ceramics are the preferred materials for the subtractive milling of crowns and bridges. Due to their superior mechanical stability, oxide ceramics are frequently selected as the material of choice for a wide range of dental applications [10]. Oxide ceramics consist of a pure polycrystalline phase without any glass phase. Today, zirconium oxide (ZrO_2_) is predominantly used, to which 3 mol% yttria (Y_2_O_3_) is added in order to stabilize the crystals in the tetragonal crystal phase at room temperature (yttria-stabilized tetragonal zirconia polycrystals, Y-TZP) [10]. At 3 mol% yttria, dental zirconium oxide ceramics (3Y-TZP) exhibit the highest fracture toughness. However, these ceramics are almost opaque due to the birefringence of the tetragonal crystals and the numerous grain boundaries [10]. They are suitable only as framework structures for single crowns or multi-unit veneered bridges, which must be veneered with silicate ceramics. The aesthetics of these veneered restorations are unrivaled. However, chipping of the veneer often occurs. An alternative to minimize fracture risk is to fabricate the restoration monolithically. Adding 4 and 5 mol% of yttrium oxide (4Y- and 5Y-TZP) decreases the proportion of zirconium oxide crystals in the tetragonal phase, and the proportion of cubic crystals and grain sizes increases. Zirconium oxide becomes translucent and more aesthetic, but fracture toughness decreases [11]. These modern 4Y and 5Y-TZP zirconium oxide ceramics with color gradients in combination with coloring techniques and dental expertise allow for monolithic restorations that are aesthetically more than satisfactory [12].

In this regard, the marginal accuracy of fit between crowns or fixed partial dentures and abutment teeth, which was defined by Holmes et al. [13] as the linear distance from the edge of the restoration to the preparation margin of the die, has been well-known for a long time, and represents an essential aspect with regard to the long-term clinical success of prosthetic restorations. The marginal measuring distance extends 1 mm in the direction of the lumen from the edge of the preparation and restoration, respectively [14,15]. The authors also determined the absolute marginal discrepancy (xyz), which results from the angular combination of the vertical and horizontal marginal discrepancy as the hypotenuse of a right-angled triangle, as the margins of fixed restorations often exhibit over- or under-extension [13]. For milled restorations, the vertical fit is influenced by the number of axes of the milling machine [16]. Inadequate crown margins can lead to gingival inflammation, which results in periodontal disease or secondary caries of the abutment tooth due to the washout of the luting material [1,17,18,19,20]. In addition, deviations in fit can cause increased stress within the restorative material, which can reduce the strength of the material and cause failure by fracture [1,21]. In the literature, previously non-evidence-based recommendations of a clinically acceptable marginal gap vary from 50 to maximum tolerance values of 120 µm under clinical conditions [22]. However, a certain amount of space is required during insertion for cementation of the restoration, and this is unavoidable [23]. At the same time, the applied luting technique and the properties of the corresponding luting materials, as well as their flow behavior during the cementation process, can influence the final size of the marginal gap [24,25,26].

Basically, a distinction can be made between luting cements and composites for the final cementation of restorations; the former can be further divided into conventional and modified luting materials. Common conventional luting materials include phosphate and glass–ionomer cements based on an acid-base reaction in which the bond is reinforced by the retention and resistance of the restorative abutment teeth by means of microretentions [27,28,29,30]. As this class of materials has evolved to expand the range of applications and improve properties, modifications have been made, resulting in the introduction of resin-modified, metal-reinforced, and high-viscosity materials. In particular, resin-modified glass–ionomer cements have entered the market as luting cements. The polymerization of these two-component materials, consisting of a photopolymerizable monomer, ionizable glasses, and water, is also based on an acid-base reaction [31,32,33,34,35]. In contrast, composites are used for the adhesive cementation of indirect restorations via both microretentions and chemical bonding. The classic representatives require conditioning of the tooth, whereas the newer, self-adhesive composites interact chemically and physically with the tooth surface [36]. Preheating composite resin for luting procedures is used to reduce material viscosity and improve restoration setting [37].

The final film thickness of the luting material is important, as failure to meet this required standard would result in poor seating of the restoration, disrupting both functional and occlusal relationships [38]. Ideally, the material that is used should be able to flow out to a low film thickness, which is influenced by various factors, such as the size and shape of the filler particles, the viscosity in the uncured state, and the setting rate [39,40]. The International Organization for Standardization (ISO) defines various standards for dental luting materials, such as requirements and test methods for powder/liquid acid-base dental cements [41], water-based resin-modified cements [42], and polymer-based materials with adhesive components [43,44]. The mentioned norms require a maximal film thickness of 25 μm for acid-base dental cements and 50 μm for resin-based luting materials [45].

However, film thickness measurements set up following the aforementioned ISO norms do not consider the effects of the geometry of the abutment and the crown on the material flow. Different studies regarding the internal fit of luted restorations can be found in the literature, but none of them have used non-destructive methods and different classes of luting materials.

Therefore, the present study investigated the extent to which different luting cements and materials influence the resulting marginal accuracies of CAD/CAM-milled zirconia single-tooth restorations after subtractive fabrication using a digital non-destructive method. The first hypothesis was that there is no difference in the fit of a particular zirconia single-tooth restoration before and after cementation. The second hypothesis was that there is no difference in the fit of the different zirconia single-tooth restorations after cementation.

## 2. Materials and Methods

The measurements carried out in this in vitro investigation were based on a metal master model, which corresponded to an in vivo chamfer preparation of a single tooth to derive an all-ceramic single crown.

Consequently, a total of 40 individual double mix impressions were taken from this master model using an addition-cured polyvinyl siloxane (AFFINIS PRECIOUS light und regular body, Coltène/Whaledent AG, Altstaetten, Switzerland), which were then poured with Class IV super hard stone (GC Fujirock EP Classic, GC, Tokyo, Japan). The individual plaster dies were digitized with a dental model scanner (Dental Wings 3SERIES, Dental Wings Inc., Montréal, QC, Canada) and DWOS 5.0.1.3084 Software (Dental Wings Inc.) used for the further CAD design of anatomically reduced zirconium oxide crown copings. During the manufacturing process, the marginal gap parameter of all crown copings was set to 20 µm, whereas the basic parameters amounted to a minimum layer thickness of 0.5 mm, a margin thickness of 0.25 mm, and vertical and horizontal placeholders for the cement of 40 μm each for all crown copings.

To three-dimensionally (3D) measure the fit between the crown copings and the plaster dies, optical object registration was performed using an ATOS Triple Scan (GOM GmbH, Braunschweig, Germany) non-contact blue-light industrial scanner. For this purpose, the plaster dies were equipped in advance with high-contrast reference points with a diameter of 0.4 mm (GOM GmbH) to enhance the precision of the subsequent scanning process. All scanning procedures were conducted by the same experienced clinician.

In accordance with the triple scan protocol of Holst et al. and Matta et al. [46,47], for each case, four single scans were taken of the corresponding dies and crown copings so that the copings could be positioned correctly on a virtual plane before and after cementation. First, all of the plaster dies and crown copings were optically scanned separately; the crown copings were coated in advance with a thin layer of a mixture of 90% ethanol and pure titanium dioxide powder using an airbrush to reduce possible light reflection, and then fixed in a specially calibrated measuring frame (Reference frame, GOM GmbH).

Subsequently, the copings on the plaster dies, which were fixed in the adapted position with adhesive wax (Supradent-Wax, Anton Gerl GmbH, Munich, Germany) before cementation, were scanned together before and after definitive cementation. The cementation procedures were performed under a constant punch pressure of 10 N in a standardized manner using a rondel construction [48,49]. All cementation procedures were performed by the same experienced clinician who had previously performed the scanning procedures.

As the dependence of the selected luting material on the fit of the crown copings was also investigated in this study, four different luting materials were selected, each cementing 10 crown copings in self-cure mode: a zinc phosphate cement (HOFFMANN’S READY2MIX ZINC PHOSPHATECEMENT NORMAL, Hoffmann Dental Manufaktur, Berlin, Germany—Group A), a glass–ionomer cement (Ketac Cem Aplicap, 3M, St. Paul, MN, USA—Group B), a self-adhesive resin cement (RelyX Unicem 2 Automix, 3M, St. Paul, MN, USA—Group C), and a resin-modified glass–ionomer luting cement (GC FujiCem 2 Automix, GC, Tokyo, Japan—Group D). The luting materials under investigation, batch numbers, composition, filler sizes, and film thickness as disclosed by the manufacturers are listed in Table 1.

**Figure 1:**
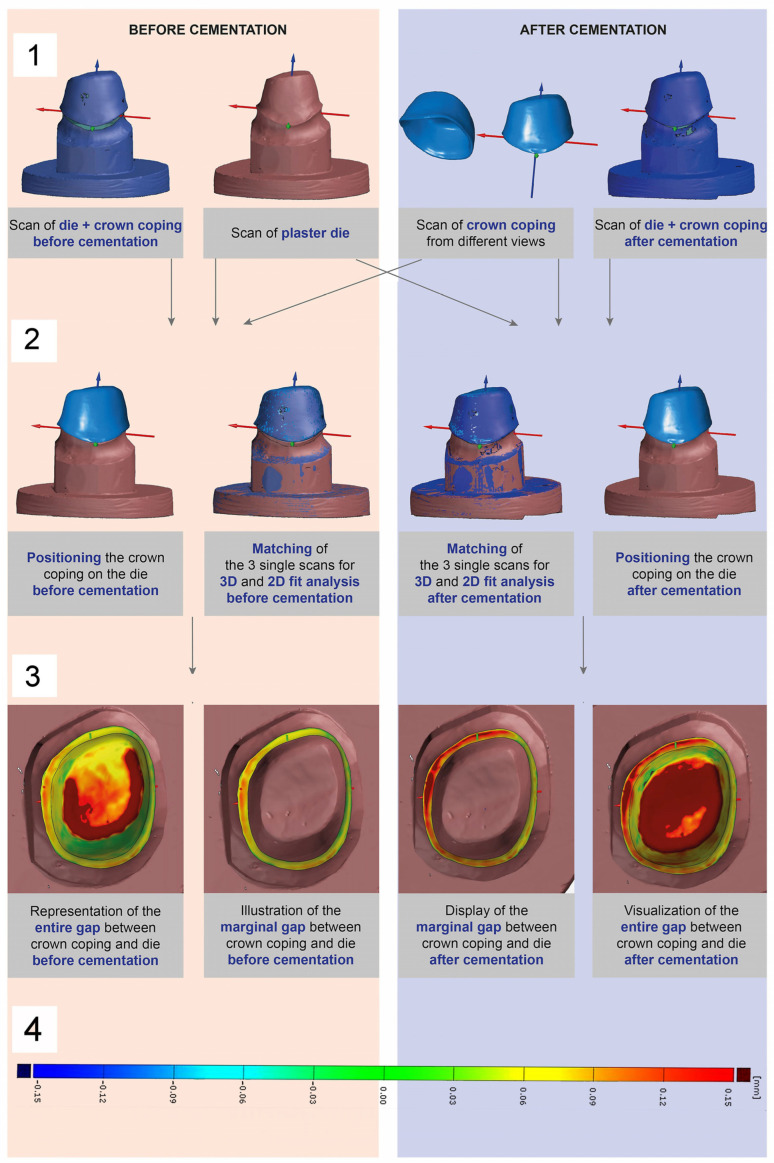
(**1**) Analytical protocol consisting of four scans in each case: single scan of the plaster die, single scan of the ceramic crown coping, scan of the adapted coping on the die in its final position before cementation, and scan of definitively cemented coping on the die. (**2**) Virtual superimposition of the individual scans (plaster dies, crown copings) with the situations before and after cementation. (**3**) Surface analysis of the entire and the marginal gap between the crown coping and the plaster die before and after cementation. (**4**) False color scale to visualize the discrepancies (mm) as color-coded distance maps. Green areas indicate deviations between 0 and 50 µm, yellow areas show deviations from 50 to 100 µm, and red areas highlight deviations of more than 100 µm.

Surface Triangulation Language (STL) file formats were generated from the obtained data, which generally exhibited an average measurement error of 3 µm due to the scanning method and virtual object registration [46]. With the aid of the GOM “Inspect Professional” software 2017 (GOM GmbH), a virtual surface and section analysis could be performed. Therefore, the individual scans of the plaster dies and crown copings were virtually superimposed on the jointly digitized dies and copings before and after cementation (“matching”) and finally aligned with high precision for data comparisons using the local best-fit function. In the following step, a marginal surface of the copings was defined, which extended 1 mm parallel to the crown margin in the direction of the lumen, so that a 3D surface analysis of the marginal fit accuracies could be performed. Subsequently, discrepancies in this area from the virtual plaster dies as reference models could be calculated by an area comparison and visualized using a color plot. Following this procedure, a surface analysis was performed for the condition before and after cementation so that the respective fits could be compared (Figure 1).

**Figure 2:**
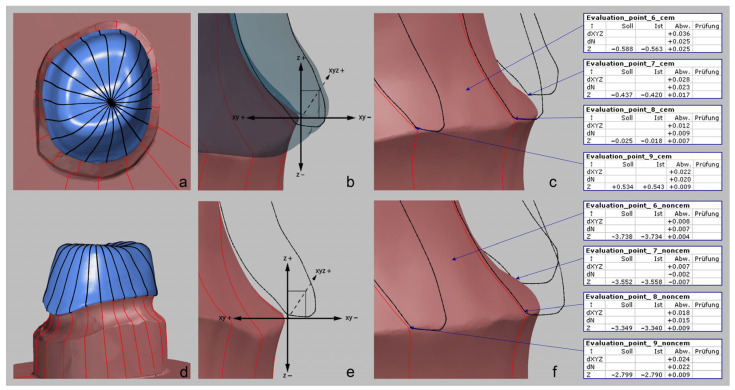
Visualization of the 2D sectional examination through the die with the 20 virtually positioned sections (**a**,**d**) and representation of the constructed coordinate system between coping and preparation margin of the plaster die (**b**,**e**). z = vertical marginal discrepancy from the most inferior edge of the coping to the outermost edge of the die, n = horizontal marginal discrepancy from the determined perpendiculars of the most inferior edge of the coping as well as the outermost edge of the die, xyz = absolute marginal discrepancy as a 2D vector of the vertical (z) and horizontal (n) discrepancy. An illustration of the 2D marginal deviations (**c**,**f**) of the coping before (**d**–**f**) and after cementation (**a**–**c**) is also provided.

In addition, a two-dimensional (2D) examination of the margin fit of the crown copings was performed, so the matched files before and after cementation were virtually split into 20 sectional images at 18° intervals. By creating a coordinate system, it was possible to calculate the vertical, horizontal, and absolute marginal discrepancies (Figure 2).

In the statistical analysis, the measurements before and after cementation of the crown copings were compared using Wilcoxon signed rank tests. The differences in the situations before and after cementation were calculated and compared between the four groups. For this purpose, a global Kruskal–Wallis test was performed and pairwise group comparisons carried out with Mann–Whitney U tests. The mean values of the 20 repeated measurements were used for the statistical tests of the 2D measurements. The statistical analysis was performed using statistical software R 4.0.3 [50] with a significance level of 0.05.

## 3. Results

**Figure 3:**
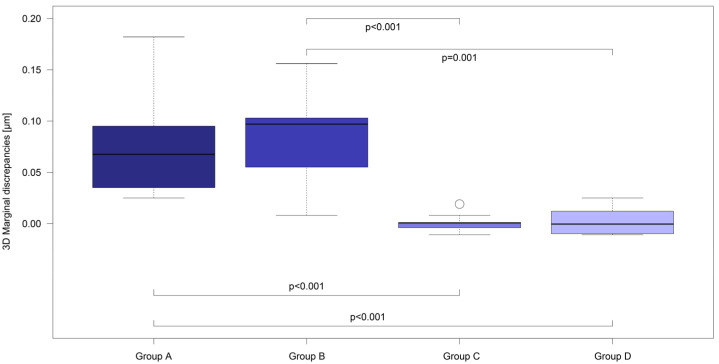
Comparison of marginal 3D differences between the four study groups (A–D) using boxplot diagrams.

The 3D measurements of the crown copings before and after cementation (Table 2), compared by Wilcoxon signed rank tests, illustrate significant differences within Group A (deviation in µm: 0.033 ± 0.004 before vs. 0.110 ± 0.049 after cementation, *p* = 0.002) and Group B (deviation in µm: 0.035 ± 0.005 before vs. 0.118 ± 0.048 after cementation, *p* = 0.002). To determine the influence of the selected luting material on the resulting fit, and thus the discrepancies between the four groups, the differences in the respective situations were calculated and compared between the groups using the global Kruskal–Wallis test and pairwise group comparisons with Mann–Whitney U tests. Group A (difference in µm: 0.077 ± 0.049) exhibited a significantly larger marginal gap (*p* < 0.001) than Group C (difference in µm: 0.001 ± 0.008), and Group D (difference in µm: 0.001 ± 0.012). In addition, a significantly larger deviation was observed in Group B (difference in µm: 0.083 ± 0.046) compared to Group C (*p* < 0.001) and Group D (*p* = 0.001; Figure 3).

In the course of the 2D examinations (Table 3) of the marginal fit at the two time points before and after cementation of the copings, significant differences were elicited in the vertical dimensions within Group A (deviation in µm: 0.030 ± 0.015 before vs. 0.193 ± 0.146 after cementation, *p* = 0.002) and Group B (deviation in µm: −0.004 ± 0.015 before vs. 0.164 ± 0.092 after cementation, *p* = 0.002). In contrast, the horizontal marginal discrepancies differed significantly only within Group B (discrepancy in µm: 0.010 ± 0.006 before vs. 0.020 ± 0.009 after cementation, *p* = 0.002).

Regarding the absolute marginal discrepancy, significant differences were established not only within Group A (deviation in µm: 0.045 ± 0.014 before vs. 0.207 ± 0.148 after cementation, *p* = 0.002) and B (deviation in µm: 0.031 ± 0. 005 before vs. 0.187 ± 0.094 after cementation, *p* = 0.002), but also within Group D (deviation in µm: 0.017 ± 0.003 before vs. 0.038 ± 0.016 after cementation, *p* = 0.002).

**Figure 4:**
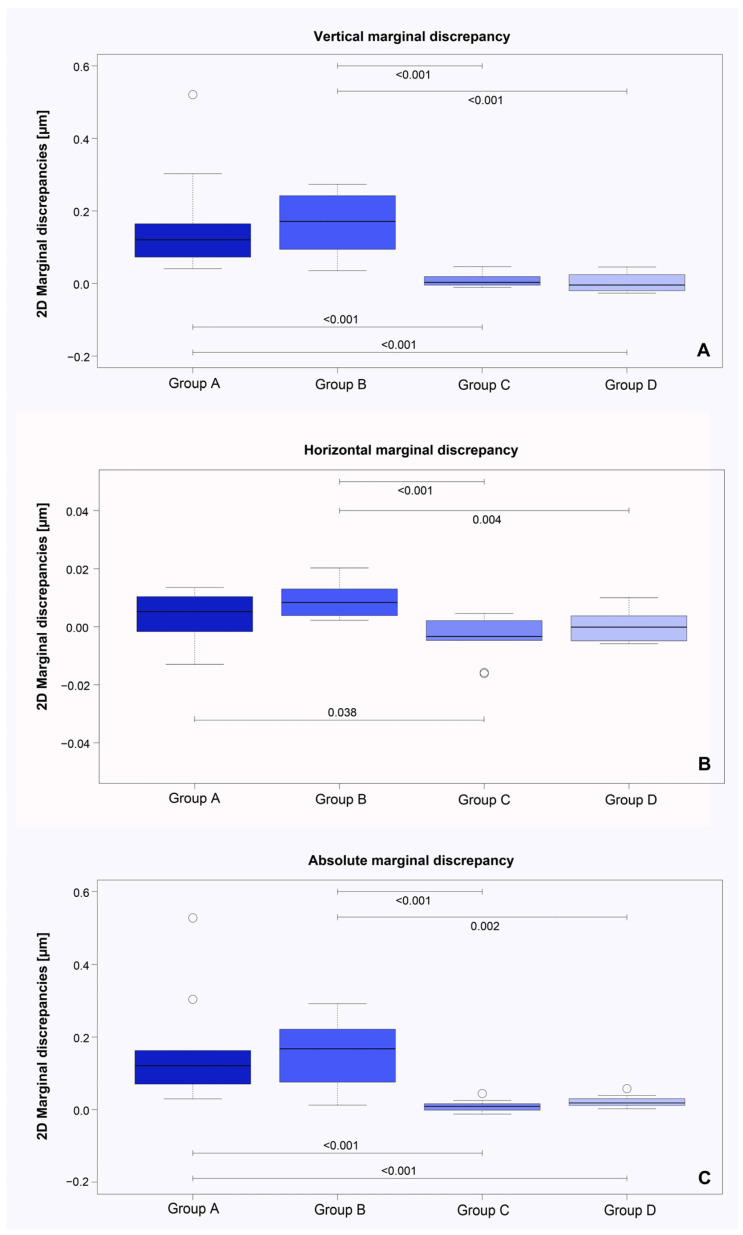
Comparison of marginal 2D differences with regard to the vertical (**A**), horizontal (**B**) and absolute marginal discrepancies (**C**) between the four study groups (A–D) using boxplot diagrams.

For the statistical comparison of the 2D measurements between the respective groups, the differences in the averaged values were used. Groups A (difference in µm: 0.164 ± 0.146) and B (difference in µm: 0.169 ± 0.087) showed significantly larger vertical marginal deviations (*p* < 0.001) than Groups C (difference in µm: 0.009 ± 0.018) and D (difference in µm: 0.002 ± 0.025). Concerning the horizontal deviations, significant differences (*p* = 0.038) were observed when comparing Groups A (difference in µm: 0.004 ± 0.008) and C (difference in µm: −0.004 ± 0.007). In addition, Group B (difference in µm: 0.009 ± 0.006) exhibited significantly larger horizontal discrepancies than Groups C (*p* < 0.001) and D (difference in µm: 0.001 ± 0.006, *p* = 0.004). With regard to the comparison of absolute marginal discrepancies between all groups, significantly larger discrepancies were found for Groups A (difference in µm: 0.162 ± 0.150) and B (difference in µm: 0.155 ± 0.095) compared to Groups C (difference in µm: 0.009 ± 0.017, *p* < 0.001) and D (difference in µm: 0.022 ± 0.017, *p* < 0.001 and *p* = 0.002, Figure 4).

## 4. Discussion

This study aimed to identify the most commonly used materials for luting macroretentive zirconia restorations in dental practice. It selected different representative materials and mixing modes for the applications. Group A involved hand-mixing zinc phosphate cement, which is widely used in dental practices due to its cost-effectiveness and long shelf life. Group B included glass–ionomer as a capsule mix material. Group C investigated the effectiveness of a gold standard self-adhesive automix resin cement, and Group D evaluated a representative resin-modified glass–ionomer cement (RMGIC). A laboratory scanner was employed to standardize the models’ digitization and produce the crowns. Using natural teeth as die material would have complicated the repeatability of preparing the same die. Therefore, gypsum replicas were manufactured instead. The laboratory scanner yielded more reproducible scan results compared to an intraoral scanner due to standardized scan paths. Conversely, the paths with the intraoral scanner can exhibit greater variability from one scan to another.

The measurement technology applied in this study was based on the virtual superimposition of STL data sets for the corresponding crown copings with the plaster dies before and after cementation as a result of optical data generation, and this enabled quantitative, non-destructive marginal 3D fitting, as well as 2D section analysis. The triple scan protocol described by Holst et al. [46] enabled a large number of measurement points to be generated, and the marginal fit could also be evaluated based on the horizontal marginal, vertical marginal, and absolute marginal fit discrepancies, ensuring reliable results of increased significance. Nevertheless, optical scanning and evaluation systems may be influenced by system-related or external aspects, such as the surface quality or the scan depth of the objects, and no absolute accuracy of the measurements can be achieved [46].

The first hypothesis was that there is no difference in the fit of a zirconia single-tooth restoration from before to after cementation within the same luting material. This hypothesis has to be partially rejected, as Group A (zinc phosphate cement) and Group B (glass–ionomer cement) had significantly increased 2D and 3D marginal fit discrepancies after cementation. As stated by Jorgensen, the thickness of zinc phosphate cement between restoration and tooth and, thus, the marginal fit of crowns is influenced by cementation pressure and duration, cement viscosity, temperature, and preparation taper influence [51]. Among all of the materials under investigation, the luting material of Group A was mixed by hand. Following the manufacturer’s recommendations, a determined powder-to-liquid ratio was mixed to obtain the cement. Despite the use of measuring aids such as scoops and dropper bottles, hand mixing has been repeatedly reported to lead to improper mixing ratios, which influences different material properties, including cement viscosity and working time [52,53]. Walton reported that significantly different film thicknesses were measured, even by experienced clinicians under standardized ambient conditions and mixing instruments, and pre-weighted liquid and powder [54]. For Group A, the measured vertical marginal discrepancy increased by 0.163 µm after cementation. Considering that the actual minimum thickness detected between teeth during occlusion (minimal interdental threshold) is 17 µm, the increase in vertical marginal discrepancy for Group A would represent significant occlusal interference [55]. Under clinical circumstances, a time-consuming occlusal adjustment would be necessary. Furthermore, a marginal gap wider than 120 µm is not recommended [22]. It is very likely that, in Group A, the problems resulting from suboptimal mixing combined with the relatively low cement gap of the restorations (40 µm) led to increased 3D, 2D vertical (z), and absolute margin discrepancies (xyz). This lack of marginal fit calls into question the suitability of zinc phosphate cement with modern high-fit CAD/CAM restorations when other luting materials are easier to apply, and achieve a better fit after cementation under the same conditions. However, in contrast to Group A, this is a glass–ionomer cement in capsule mix form. In this case, mixing-induced problems in viscosity and setting properties cannot be responsible for the increased marginal discrepancies. The restorations in the present study have a relatively narrow cement gap, resulting in a tight fit (between 0.033 and 0.042 µm 3D marginal fit). The literature shows that tighter-fitting restorations will have increased vertical lift; in the present case, there was an increased vertical marginal discrepancy (0.160 µm for Group B) [56]. The tighter fit, in combination with the probably too-high viscosity of the glass–ionomer, may have reduced the material’s outflow and led to the restoration tilting, resulting in the significantly increased horizontal marginal discrepancy.

Only Group C (self-adhesive resin cement) and Group D (resin-modified glass–ionomer cement, except for the absolute marginal discrepancy) had any influence on the marginal fit. Both materials are delivered in automix canulae, where the catalyst and base paste are mixed. Mixing the pastes in an automix syringe requires optimized rheology, which results in cementation without affecting the marginal fit [57]. The self-adhesive resin luting material can be recommended for clinical use in terms of marginal fit. In addition, the self-adhesive cement generated the most robust adhesion to zirconia among all of the luting materials under investigation due to chemical bonds between its phosphomethacrylates and the zirconia and tooth substrates [58,59,60]. The investigated resin-modified glass–ionomer was superior to the zinc phosphate and glass–ionomer cement in regard to marginal fit. However, some older resin-modified glass–ionomers were prone to swelling and hydrolytic degradation due to their hydrophilic monomer content [61,62]. Whether this applies to the modern representatives of this material class needs to be investigated.

Despite the productive and interesting findings, it is imperative to acknowledge the limitations of the current study. Although a representative selection of fastening materials was made, incorporating a substantial portion of those currently available, it only encompasses a fraction of the variety accessible today. Additionally, factors such as the geometry of the tooth die, i.e., the convergence angle, the inner transitions, and the design of the preparation margin, may influence cement flow behavior. Consequently, while the study’s results remain valid, they should be interpreted in light of these considerations.

The results of the present study suggest that the influence of luting materials on the fit of restorations is a complex issue. All investigated materials meet their respective ISO norms, especially for film thickness. However, the ISO method does not seem to be able to offer absolute conclusions about how the fit of the restoration is ultimately influenced. As can be seen from the literature, a large number of factors, in addition to the film layer thickness, are involved. Investigating and standardizing these influencing factors (convergence angle, preparation margin, cement, and margin gap) should be the subject of further investigations to precisely match the properties of the luting materials and thus achieve a perfect fit. The method used here may be helpful for this purpose.

## 5. Conclusions

Within the limitations of the present study, the authors drew the following conclusions:

- The digital non-destructive method was able to detect the influence of the luting material on the fit of a zirconia single-tooth restoration before and after cementation;
- The zinc phosphate cement led to increased vertical marginal discrepancies;
- Only the self-adhesive luting resin did not influence the fit of the restoration after cementation, and can be clinically recommended.

## Figures and Tables

**Table 1:** Manufacturer’s specifications of the luting materials investigated.

Luting Material	HOFFMANN’S READY2MIX NORMAL	Ketac Cem Aplicap	RelyX Unicem 2 Automix	GC Fuji-Cem 2
Material type	Zinc phosphate	Glass- ionomer	Self-adhesive resin	Resin-modified glass–ionomer
Manufacturer	Hoffmann Dental Manufaktur GmbH, Berlin, Germany	3M, St. Paul, MN, USA	3M, St. Paul, MN, USA	GC, Tokyo, Japan
Shade	Yellow	Yellow	A2	Light Yellow
Lot number	N.A.	529059	574731	141211A
Composition	Powder: Zinc oxide, magnesium oxide Liquid: Ortho-phosphoric acid	Powder: Glass powder, pigments Liquid: Water, Acrylic acid/Maleic acid copolymer, tartaric acid, preservative	Base paste: Phosphorylated methacrylate monomers, methacrylate monomers, silanized fillers, initiators, stabilizers, rheology additive Catalyst paste: Methacrylate monomers, basic and silanized fillers, initiators, stabilizers, pigments, rheology additive	Paste A: Fluoroalumino- silicate glass, initiator, UDMA, dimethacrylate, pigments, silicon dioxide, inhibitor Paste B: Silicon dioxide, UDMA, dimethacrylate, initiator, inhibitor
Filler particle size	N.A.	≤12 µm	<9.5 µm	N.A.
Film thickness	N.A.	16 ± 1 µm	13 µm	N.A.

**Table 2:** Descriptive statistics of the 3D measured values of the marginal fit in µm, the corresponding standard deviations (SD), the maximum and minimum values (Max, Min) and the *p*-values when comparing the two time points before and after cementation for all groups (Group A–D).

3D Analysis of the Marginal Fit (µm) before and after Cementation						
Group		Mean	SD	Min	Max	*p*-Value
Group A	before cem	0.033	0.004	0.028	0.038	0.002
	after cem	0.110	0.049	0.059	0.210	
Group B	before cem	0.035	0.005	0.031	0.043	0.002
	after cem	0.118	0.048	0.039	0.188	
Group C	before cem	0.042	0.005	0.035	0.053	1.0
	after cem	0.042	0.007	0.033	0.058	
Group D	before cem	0.038	0.003	0.035	0.042	0.722
	after cem	0.040	0.012	0.027	0.067	

**Table 3:** Mean values of the data of the 2D virtual analysis of the vertical, horizontal and absolute marginal discrepancy in µm before and after cementation for all groups (Group A–D). Furthermore, representation of the calculated standard deviations (SD), the maximum and minimum values (Max, Min) and the *p*-values when comparing the respective data series.

2D Analysis of the Marginal Fit (µm) before and after Cementation							
Parameter	Group		Mean	SD	Min	Max	*p*-Value
Vertical marginal discrepancy	A	before cem	0.030	0.015	−0.002	0.052	0.002
		after cem	0.193	0.146	0.051	0.551	
	B	before cem	−0.004	0.015	−0.025	0.017	0.002
		after cem	0.164	0.092	0.011	0.291	
	C	before cem	0.018	0.007	0.008	0.031	0.322
		after cem	0.026	0.020	−0.003	0.064	
	D	before cem	0.011	0.002	0.007	0.014	0.846
		after cem	0.013	0.025	−0.014	0.057	
Horizontal marginal discrepancy	A	before cem	−0.021	0.014	−0.049	0.002	0.232
		after cem	−0.017	0.019	−0.046	0.015	
	B	before cem	0.010	0.006	0.003	0.024	0.002
		after cem	0.020	0.009	0.006	0.038	
	C	before cem	0.018	0.005	0.009	0.026	0.126
		after cem	0.014	0.006	0.008	0.026	
	D	before cem	0.012	0.003	0.009	0.017	0.922
		after cem	0.013	0.006	0.006	0.023	
Absolute marginal discrepancy	A	before cem	0.045	0.014	0.031	0.076	0.002
		after cem	0.207	0.148	0.076	0.570	
	B	before cem	0.031	0.005	0.026	0.042	0.002
		after cem	0.187	0.094	0.048	0.319	
	C	before cem	0.028	0.009	0.014	0.041	0.131
		after cem	0.037	0.016	0.021	0.070	
	D	before cem	0.017	0.003	0.012	0.023	0.002
		after cem	0.038	0.016	0.021	0.075	

## Data Availability

The data sets used and/or analyzed during the current study are available from the corresponding author upon reasonable request.

## Abbreviations

CAD	Computer-Aided Design
CAM	Computer-Aided Manufacturing
3D	Three-dimensional
2D	Two-dimensional
STL	Standard Transformation Language
Mean	Mean distance
SD	Standard deviation
Min	Minimum distance
Max	Maximum distance
